# P-678. Comparison between the Characteristics and Outcomes of Patients Hospitalized for COVID-19 in Three Waves of the Pandemic

**DOI:** 10.1093/ofid/ofae631.874

**Published:** 2025-01-29

**Authors:** Barbara Mares Porto, Daniella Nunes Pereira, Luciane Kopittke, Alisson Asevedo, Angélica Gomides dos Reis Gomes, Angelinda Behring, Beatriz Lima, Carla Silva, Cláudia Barros, Elayne Pereira, Evelim Thainara Conceição de Oliveira, Flavia Vigil, Gabriela Crestani, Lais Santos, Leila Beltrami Moreira, Marco Reis, Maria Bicalho, Vanessa Muller, Unaí Tupinambás, Milena Soriano Marcolino

**Affiliations:** Universidade Federal de Minas Gerais, Belo Horizonte, Minas Gerais, Brazil; Universidade Federal de Minas Gerais, Belo Horizonte, Minas Gerais, Brazil; Hospital Nossa Senhora da Conceição, Porto Alegre, Rio Grande do Sul, Brazil; Hospital Santa Rosália, Teófilo Otoni, Minas Gerais, Brazil; Hospitais da Rede Mater Dei., Belo Horizonte, Minas Gerais, Brazil; Hospitais da Rede UNIMED-BH, Belo Horizonte, Minas Gerais, Brazil; Orizonti - Instituto de Saúde e Longevidade, Belo Horizonte, Minas Gerais, Brazil; Hospital Santo Antônio, Curvelo, Minas Gerais, Brazil; Hospital Universitário Professor Edgard Santos, Salvador, Bahia, Brazil; Hospital SOS Cárdio, Florianópolis, Santa Catarina, Brazil; Hospital Universitário Canoas, Canoas, Rio Grande do Sul, Brazil; Hospital Metropolitano Doutor Célio de Castro, Belo Horizonte, Minas Gerais, Brazil; Hospital Mãe de Deus, Porto Alegre, Rio Grande do Sul, Brazil; Hospital Santa Cruz, Santa Cruz do Sul, Rio de Janeiro, Brazil; Hospital de Clínicas de Porto Alegre, Porto Alegre, Rio Grande do Sul, Brazil; Hospital Risoleta Tolentino Neves, Belo Horizonte, Minas Gerais, Brazil; Universidade Federal de Minas Gerais, Belo Horizonte, Minas Gerais, Brazil; Hospital São Lucas, Porto Alegre, Rio Grande do Sul, Brazil; Universidade Federal de Minas Gerais - UFMG, Belo Horizonte, Minas Gerais, Brazil; Universidade Federal de Minas Gerais, Belo Horizonte, Minas Gerais, Brazil

## Abstract

**Background:**

Throughout the COVID-19 pandemic, new SARS-CoV-2 variants emerged, each prevailing during a certain time period and defining the pandemic waves that were observed all over the world. Even in different countries, such waves presented many similarities, such as the period of occurrence, clinical characteristics of those who were infected by the SARS-CoV-2 virus, COVID-19 outcomes and governmental public policies to stop the spread of the disease. Their major differences are believed to be related to the SARS-CoV-2 variant circulating at the time, as well as the availability of vaccines, patient vulnerabilities, resource availability and knowledge in patient management.

**Figure:**
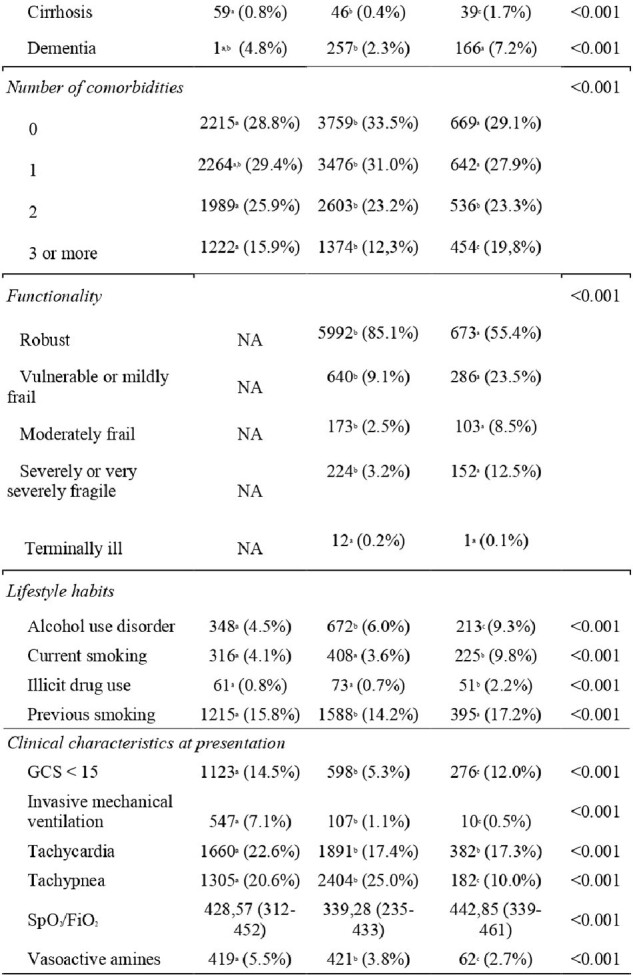

**Methods:**

This study is part of a multicentric retrospective cohort, entitled the Brazilian COVID-19 Registry, which included adult patients (≥18 years), with laboratory-confirmed COVID-19, admitted between March 2020 and August 2022 in 41 hospitals in six Brazilian states. The primary outcome was in-hospital mortality. Secondary outcomes included: intensive care unit (ICU) admission, need for mechanical ventilation, kidney replacement therapy (KRT) thromboembolic event, nosocomial infection, acute heart failure and septic shock.

**Results:**

The median age decreased in the second wave and increased in the third compared to the first. The total number of comorbidities was higher during the third wave and lower in the second. When evaluating symptoms, in the third wave, anosmia, ageusia and fever were less common when compared to the other waves , while neurological manifestations were more frequent. The frequency of admission to the intensive care unit and in-hospital mortality reduced throughout the pandemic.

**Conclusion:**

The study showed that clinical manifestations and outcomes changed between the three COVID-19 waves. In the second phase of the pandemic, patients hospitalized with Covid-19 were younger and had fewer comorbidities. The risk of severe disease has decreased substantially since the start of the pandemic. However, the new strains are under continued investigation, because of the unknown impacts in severity and vaccine effectiveness, the response to existing therapies and the accuracy of diagnostic tools.

**Disclosures:**

**All Authors**: No reported disclosures

